# Invasive raccoon management systems and challenges in regions with active control

**DOI:** 10.1186/s12898-020-00336-0

**Published:** 2020-12-11

**Authors:** Takaaki Suzuki, Tohru Ikeda

**Affiliations:** 1 Graduate School of Letters, Hokkaido University, Kita 10 Nishi 7, Kita-ku, Sapporo, Hokkaido 060-0810 Japan; 2 National Institute for Environmental Studies, 16-2 Onogawa, Tsukuba, Ibaraki 305-8506 Japan

**Keywords:** Biological invasion, Raccoon, Management, Biodiversity monitoring, Non-native species

## Abstract

**Background:**

The raccoon (*Procyon lotor*) is an invasive, non-native species in Japan. Throughout the country, it causes significant agricultural damage and negatively affects native biodiversity. Most of the responsibility for raccoon management lies with local government, and there are still many challenges to be overcome. Although raccoon populations have not been eradicated, intensive control campaigns such as focus on the early stages of invasion have controlled raccoons in some regions. To improve the national management of raccoons, we conducted a survey on raccoon management systems in local government departments considered to solve the challenges recognized in many areas. During 2014 and 2015, we surveyed three different municipal departments about raccoon management measures. The semi-structured interview survey covered two topics: (1) the situation leading up to the current management system; (2) the current management system.

**Results:**

Our results describe the scope and methods used in raccoon management. The government staff managed raccoons using monitoring, employing a variety of methods, a range of budgets, and various role divisions. The management practices are similar in that they share a sense of taking precautions, collaborating with stakeholders, understanding that adequate methods must be used, and obtaining support from experts.

**Conclusions:**

Our case studies reveal the challenges in raccoon management faced by local government officers in regions with active control. The management systems and methods that we surveyed seemed to be effective in solving problems in both developed and undeveloped areas.

## Introduction

Invasive non-native species are recognized internationally as a significant threat to indigenous biodiversity [1–6]. Once invasive species have become established, they must be managed by sustained control programs or, if achievable, eradicated [5, 7]. Government-led invasive alien species (IAS) control programs have been initiated and critically evaluated in countries like Australia and New Zealand [8, 9]. In such programs, although the effects of management interventions (such as pest reduction) are usually monitored, the outcomes (such as increases in biodiversity), are not [8, 9]. Planning and implementing well-designed and well-monitored IAS programs is a global challenge [8–11].

In recent years, interdisciplinary research about invasive species has increased; however, many of these studies focus on ecological themes [12, 13]. Many of the studies that use social surveys address the psychology, ethics, and conceptual themes related to invasions [14–16], but few studies deal directly with management practices. It is known that there is a gap between research and management practice [17–20]. To address this gap, researchers must have a detailed understanding of the situation in the field. On the other hand, although there are many successful examples of management, these are difficult communicate to the public as they are rarely published [21]. Even when they have been documented, these examples may not be sufficient for policy development [22]. Research is required not only on the various environmental aspects of IAS management [23], but also on social aspects [22]. For example, Head et al. [22] mentioned that the experiences of managers are worth documenting, because they offer critical insights into the basis of contemporary priority setting and pragmatic decision making. There are few studies that explain why this problem exists and how to solve it; such information is required for reference in developing management plans.

The common raccoon is a mammal native to North America [24–26] that has been introduced to many countries; it is widespread in Europe and partly in Russia, West Asia and Central Asia [27]. In Japan, escapes from captivity and irresponsible releases led to the first reports of raccoon naturalization in Inuyama, Aichi Prefecture, in 1962 [28, 29]. Today, raccoon invasions have become a nationwide problem [30] and they have become naturalized in most of the 47 Japanese prefectures [31, 32]. Raccoons cause damage to native ecosystems. For example, raccoons prey on Japanese crayfish (*Cambaroides japonicus*) [33], cause economic losses in agriculture (to the extent of more than 320 million yen in 2017 in Japan) [34], and damage property, including important cultural assets such as shrines and temples [35]. They also spread zoonotic diseases such as raccoon roundworm [36]. Before raccoons were given the IAS designation, they were caught as part of nuisance control programs, because of the considerable damage they cause to agriculture. The raccoon has been designated an IAS according to the Japanese Invasive Alien Species Act. This has led to the introduction of raccoon management programs.

A raccoon management program has been implemented, based on legislation regarding the hunting and control of harmful birds and mammals (specifically, the Wildlife Protection and Hunting Management Law [37]. Since it was developed as a measure against damage to agriculture and property [38], the local municipalities in charge of the cities, towns, and villages are responsible for these control programs. Cooperation between and among prefectures (the largest administrative divisions of Japan) and municipalities is needed if raccoons are to be managed effectively across Japan. However, prefectural and municipal governments manage raccoons independently of each other. Although the Ministry for Environment has issued manuals about raccoon management (e.g., [39, 40]), many contiguous local governments neither cooperate nor share information about raccoon management, even about basic methods, outcomes, or lessons that can be learnt [41, 42]. Previous studies have shown that intensive control during the early invasion stage can control raccoons effectively [38, 43, 44]. However, few regions have managed to control raccoons effectively, and they have not been eradicated anywhere in Japan.

The basic procedure for raccoon management is as follows (first version: [39]; revised version: [45]): I dissemination of information and awareness raising; II understanding the spatial scope of the problem, and the damage caused; and III planning and control; step III involves i selecting the control method; ii establishing accountability and consensus for control; iii conducting trapping; iv monitoring of the raccoon population; and v reviewing the plan (these steps form a management cycle). In many places, control measures are introduced only after damage to crops has occurred [30, 46]. An example of such control measures in response to crop damage is to repeat the passive procedural steps described earlier, in which the municipalities receive damage reports from the residents, lend cage traps to the affected residents, and the raccoons are captured. There are few local municipalities in which monitoring is implemented after trapping, and quantitative effect indicators are rarely set, so adaptive management has not been used effectively [11]. However, some areas have implemented the necessary monitoring measures for adaptive management, and solved the widespread problems associated with raccoon management. Therefore, we focus on municipalities in which all procedure steps can be implemented. A useful approach to solving the challenges faced by many areas is to investigate how municipalities start and conduct raccoon management programs, and to study the systems that they use. In particular, it is necessary to investigate the management methods used; the systems from disseminating information and raising awareness among the public regarding post-trapping monitoring; the relationships between municipal officers, raccoon experts, and program managers. The Ministry of the Environment also recommends that management case studies, including results and outcomes, be shared [41, 42]. Case studies that have resolved problems have common characteristics. Such sharing enables municipalities to learn how to cope with the challenges of raccoon control programs before they begin. In this paper, we aim to elucidate common denominators of successful raccoon management. Comparing case studies we also reveal active raccoon management systems and the challenges faced by local government officers in terms of their relationships with stakeholders.

## Materials and methods

This study received ethical approval from the research ethics committee of the Graduate School of Letters, Hokkaido University, Japan. From May 2014 to February 2015, we conducted semi-structured interviews and a participant observation survey to obtain in-depth perspectives on the daily practices of local government officers in related departments. These departments include those of Oita City, Oita Prefecture, Asahikawa City, Hokkaido, Hadano City, and Kanagawa Prefecture. These departments carry out management procedures from steps I–III (see "Introduction"). Only a few of these departments are in low-density raccoon regions. These include Oita city; Asahikawa city, which has carried out over 1000 trap nights per year, and which is in the second prefecture in Japan to be invaded; and Hadano City, which is in the third prefecture to be invaded. Further, the quantitative indices for management were developed in Hadano City. During the survey, we investigated situations regarding management procedures, stakeholder roles, the situation leading up to the current management system, and the reference information available to officers. As a basic procedure, we first conducted an interview about the subjects mentioned above in the office for over an hour. Next, I accompanied the officer to the site and conducted a survey while receiving explanations. Finally, based on the on-site situation, further interviews were conducted at the office for a sufficient amount of time until saturation. Survey answers were written down immediately. In each area, we also interviewed the stakeholders involved in raccoon management. These stakeholders included residents, environmental conservation NPOs in Oita City, the forestry association and trapper association in Asahikawa City, and, in Hadano City, a raccoon control expert allocated by the prefecture.

We aimed to understand racoon management efforts made in each region, and their associated challenges, by interviewing officers in municipalities that have different budgets, in several prefectures. Their departments handle various tasks; however, we focus on those related to raccoon management. In our case study, we divided the efforts into the situation leading up to the current management system, and the current management system. The latter incudes the reference information that the officers use, and information they would find useful for improving management programs. Further, we discuss the features of each area, and how raccoon management could be implemented in these places, considering the efforts required and the challenges faced by officers.

## Results

We conducted interviews with a total of 13 people multiple times (Table 1). Surveys were carried out in the local government’s office and on site during the trapping campaign and/or while dealing with reports from residents.

**Table 1:** Interviewees and total interview time of each interviewee in each area

Area	Interviewee	Total interview time per one person
Oita city	Two officers	More than 4 h
	One NPO president	More than 4 h
	One resident	Approximately 30 min
Asahikawa city	One officer	More than 4 h
	Four trappers	Approximately 1.5 h
	Two forestry association staff	Approximately 30 min
Hadano city	One officer	More than 4 h
	One expert	More than 4 h

### The situation leading up to the current management system

In Oita city, footprints suspected of being raccoon were found on the coast around a sea turtle landing area in 2011. These prints were later identified as not belonging to raccoons, but raccoon invasions in the city were recognized from other sources such as road killing. Because the number of the sea turtle landings and eggs has decreased over time, researchers have studied the effects of non-native species on the turtles. A local environmental conservation (NPO) (hereinafter referred to as NPO), concerned about the predation of sea turtle eggs, has held meetings and, together with scientific researchers, has carried out public awareness-raising activities about raccoons. As a result of these activities, awareness about raccoons has increased among residents and local government departments. In 2013, the NPO and local governments conducted localized raccoon control activities within the city, leading to the current management system.

In Asahikawa city, although habitat information was available, including information about capture by trapping, and crop damage, there was a lack of information about raccoon populations and distribution throughout the city, and an officer thought that it was necessary to investigate. The officers got a chance to use the 2011 Emergency Job Creation Program (for which about 80,000 US dollars was budgeted) to carry out trapping. During the program, raccoons were captured throughout a large part of the city, in greater numbers than expected. This caused a sense of crisis regarding raccoons in the division to which the officer belonged. In addition to obtaining the help of forestry associations and environmental conservation organizations, officers established a management group, and implemented raccoon management using the budgetary subsidies from multiple fiscal years, starting with the 2012 fiscal year.

In Kanagawa Prefecture, since 2006, the raccoon management plan has been set up at the level of prefectural government, and each municipality has applied this plan. In the second stage of this plan, Hadano City was regarded as being at the forefront of spreading raccoon distributions in the prefecture, and management was required. In 2011, the local government department created a new division to deal with non-native species. Before then, there were no facilities and equipment; these were gradually ordered and bought. In 2013, this department formed a trap–patrolling team, and conducted a habitat survey throughout the city, leading to the current management system.

### The current management system

In Oita City, the Environmental Division was responsible for raccoon management. One managing officer (working together with several officers in the same group), was provided with a budget of about 20,000 US dollars at the beginning of 2013. This budget was increased in 2014 to about 60,000 US dollars. The raccoon trapping campaign was carried out in a specific part of the city that was considered to be the core breeding area. It was said to have a high raccoon population, based on sightings, damage, and capture information; however, no information was available for the surrounding areas. Officers from the Environmental Division, as well as residents, the NPO, and members of the university were involved in the trapping campaign. This departmental division was responsible for handling sighting and damage reports from residents. The stakeholders and their main roles, which include the trapping campaign and reporting by residents, are shown in Table 2.

**Table 2:** Stakeholders in the raccoon trapping campaign Oita city (Japan), and their respective roles

Stakeholders	Main role
Local government	Create strategy; set up chain of command; plan the required tasks; communicate tasks to residents; (dissemination and awareness rising, response to reports from residents)
Residents	Trap patrolling
NPO	Habitat research; trap patrolling
University	Scientific knowledge; assess methods; advise about results

Roles not related to the trapping campaign are noted in brackets

**Fig. 1:**
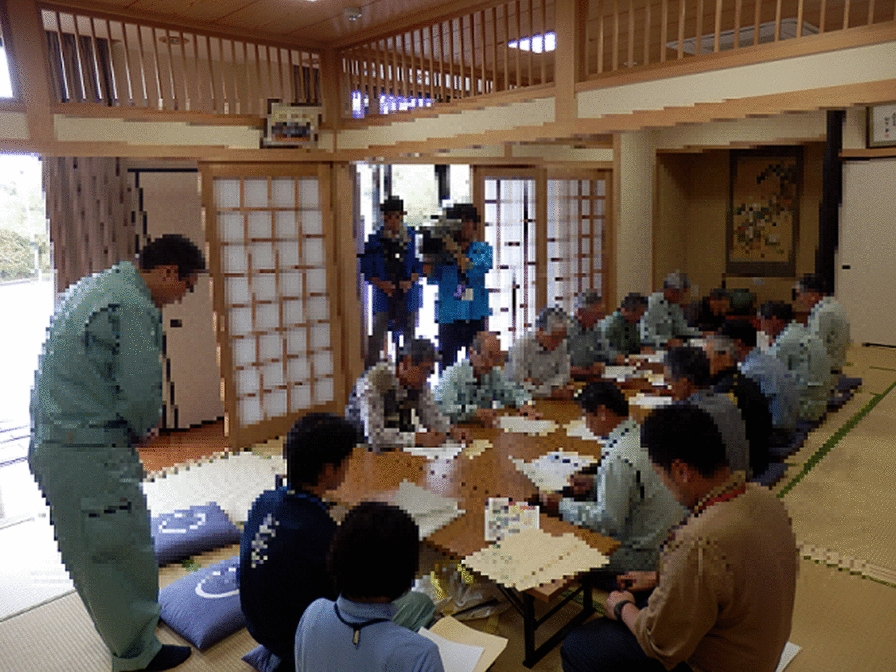
An explanatory meeting for the raccoon trapping campaign. Local government meetings will be conducted for people involved in the raccoon trapping campaign, such as residents. The organizers have notified the media in advance, and may also give interviews

**Fig. 2:**
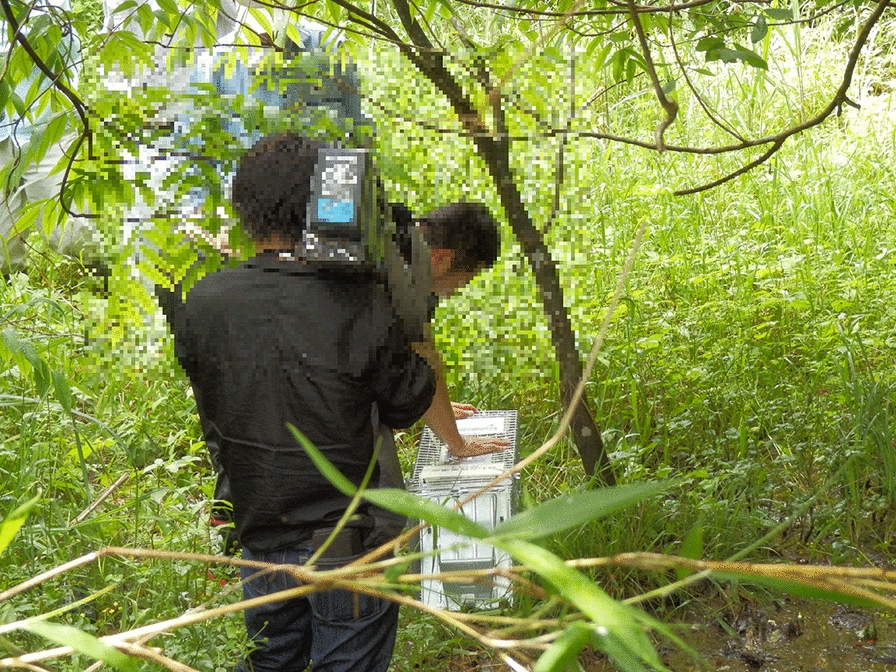
Raccoon trap setting in Oita after the meeting shown in Fig. 1. Local government guidance is given to residents who are responsible for patrolling the traps

The main implementation items in the raccoon management procedure are shown in Table 3. Raccoon management involved the following procedure: (I) Dissemination of information and awareness raising was implemented by the local government department, which provided information via a webpage, posters, leaflets, media usage, and workshops about the raccoon issue. (II) During the trapping campaign, efforts were made to understand the spatial distribution of raccoons, and damage caused; (III) Planning and control measures were implemented. Steps II and III i, used to select the control method (e.g. selection of trapping sites), were conducted by the NPO and university, using camera traps and a listening survey. During step III ii, briefings on accountability and consensus for the control process were held, mainly by local government (Figs. 1 and 2). The university provided expert knowledge regarding the material discussed at these briefings. During step III iii, traps were set and patrolled by residents and members of the NPO and local government. During steps III iv and v, debriefing meetings were held, involving all stakeholders, and camera traps were set up. The trapping campaign was conducted four times between 2013 and September 2014; 20 traps were used over a period of 2 weeks, and 16 raccoons were captured in 2013, during three trapping campaigns. From the trapping campaigns and from other reports, approximately 50 raccoons were captured each year throughout the city.

**Table 3:** Implementation measures within the procedures of the raccoon trapping campaign in each city, Japan

Procedure	Implementation measures		
	Oita city	Asahikawa city	Hadano city
I	Provide information via Web pages, posters, leaflets, and mass media	Provide information via Web pages; hold a workshop	Provide information via Web page, posters, and leaflets
II	Camera trapping; listening survey	Listening survey; last year’s capture data	Last year’s capture data; camera trapping; bait trapping; trace surveys
III i	Select trapping location	Select trapping location	Select trapping location
III ii	Hold explanatory meetings	Hold workshop	Announcing the control situation
III iii	Set traps and conduct patrols	Set traps and conduct patrols	Set traps and conduct patrols
III iv	Camera trapping; listening survey	Listening survey; capture data	Camera trapping; capture data; bait trapping; trace survey
III v	Select sites and season for trapping; select priority area	Select sites for trapping	Selecting priority areas for trapping

Damage, sighting, capture, and roadkill reports from residents were handled by the department. Departmental activities included recording the report information, reading the contents of the report, treating reports as field research, explaining the control situation to residents, and setting camera traps and cage traps.

In Asahikawa City, the Environmental Planning Division was responsible for raccoon management. Two officers were responsible, and one of them was main person in charge. The budget was approximately 40,000 US dollars. Following the Emergency Job Creation Program, these officers implemented a project named Promote Management against Non-native Species (funded by the Promotion of Biodiversity Conservation Grant: FY2012–FY2014). This grant was held by a group consisting of local government, the forestry association, and two environmental conservation organizations. Crop damage reports comprised most of the reports from residents; these were handled by the agricultural division. The environmental division was contacted only when the raccoon was captured using traps that had been set following damage reports, and the number of captures was recorded. The stakeholders and main roles are shown in Table 4.

**Table 4:** Stakeholders and their roles in the raccoon trapping campaign in Asahikawa city, Japan

Stakeholders	Main role
Environmental division of local government	Planning, determining budget, organizing groups, dissemination and raising awareness; (dissemination and awareness rising)
Forestry association	Providing a place to store equipment; management and support for the TC
Environmental conservation organization	Advisory role
Group	Review of the trapping campaign; hosting workshops
Trapper	Conducting listening surveys; setting, managing, and patrolling traps; collecting captive raccoons
Agricultural division of local government	(Responding to reports from residents)

Roles not related to the trapping campaign are noted in brackets

**Fig. 3:**
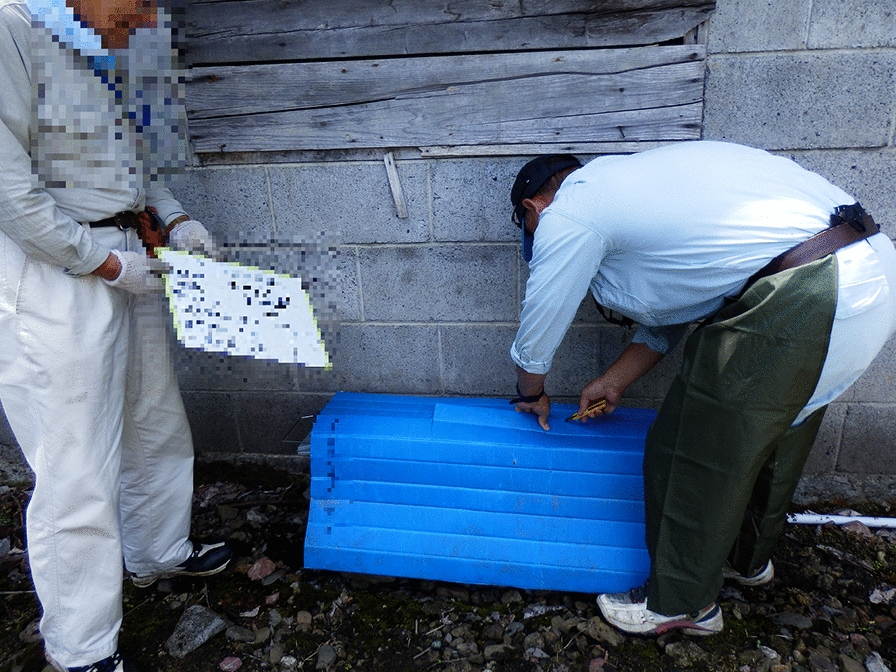
Campaign members patrolling the raccoon traps in Asahikawa. In the car during patrolling, members discuss their thoughts about the capture and management situation

The main implementation items in the raccoon management procedure in this division are shown in Table 3. Trap setting and patrolling were done by trappers (Fig. 3). Although camera traps were not used for monitoring, cage trapping was conducted throughout almost the entire city. The management group met to review the trapping campaign, based on the capture results of the relevant fiscal year. Moreover, there were opportunities for conversations between officers, forestry association members, and capture staff, and information about the raccoon control situation was discussed. In approximately 4 months, 43 traps were set. From these, 39 raccoons were captured in 2012, and 47 were captured in 2013.

In Hadano City, the Environmental Conservation Division was responsible for raccoon management. One person was responsible, and the budget was approximately 2000 US dollars. The trapping campaign program included a local government official, a wildlife damage control expert (a graduate student belonging to an official prefectural organization), a trap-patrolling team, and a team to deter monkeys. Crop damage was handled mainly by Japan agricultural cooperatives (JAs), which loaned out and set up traps for farmers. As crop damage reports were usually sent to the JAs, almost no reports were sent to the local government department. The main roles are shown in Table 5.

**Table 5:** Stakeholders and their roles in the raccoon management procedure, Hadano city, Japan

Stakeholders	Main role
Local government	All work related to the trapping campaign; trace surveys; euthanasia; (dissemination of information and raising awareness)
Expert	Advising about survey methods; supporting the trapping campaign
Trap patrolling team	Trap patrolling
Driving away the monkey team	Trap patrolling
Japan agricultural cooperative	Setting traps to reduce crop damage; (responding to reports from residents)

Roles not related to trapping campaign were noted in brackets

**Fig. 4:**
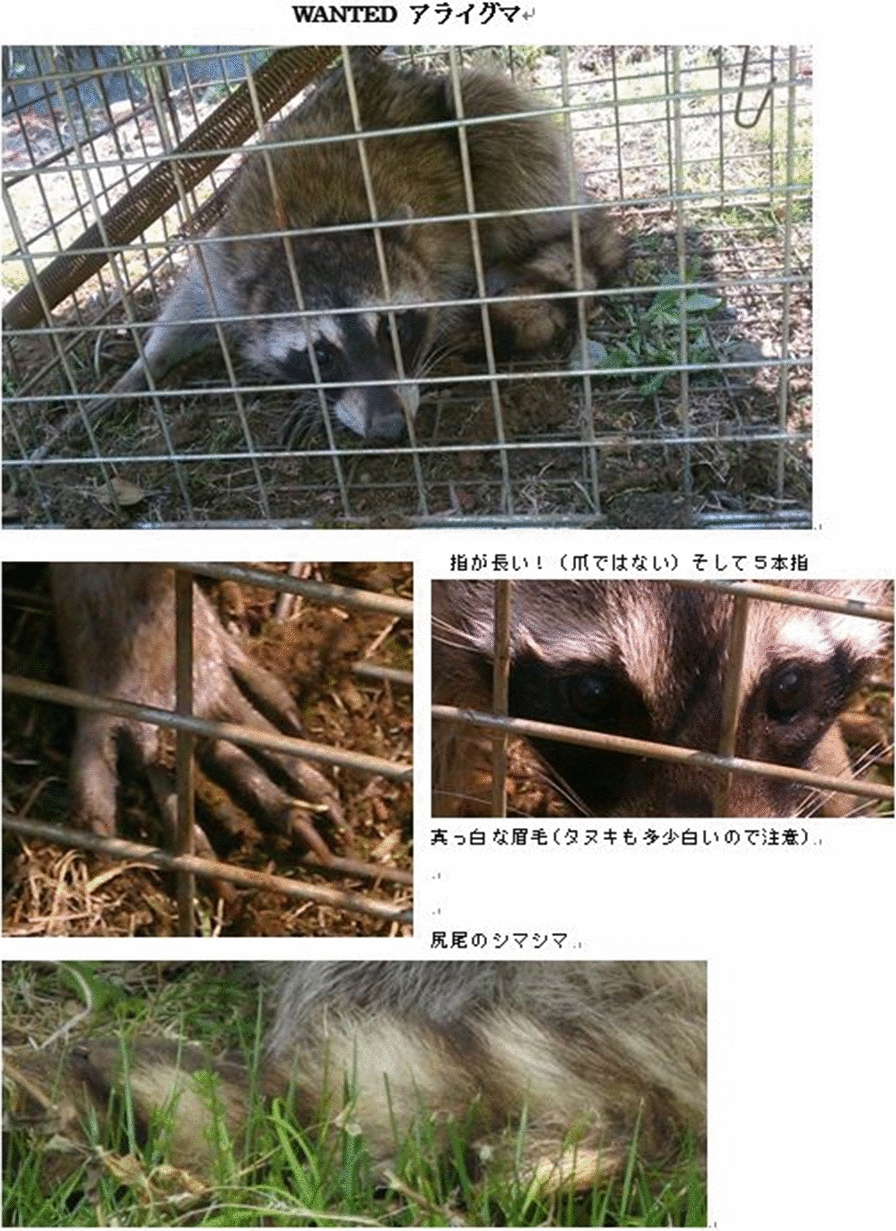
A leaflet created by an officer, to be used in the raccoon trapping campaign. The officer has also created leaflets as about the native raccoon dog (*Nyctereutes procyonoides*), which is similar to the raccoon. To prevent misreporting, this officer included photographs of other species. The leaflets were used to disseminate information and raise awareness. These leaflets are available from the city facilities and Japan Agricultural Cooperatives

**Fig. 5:**
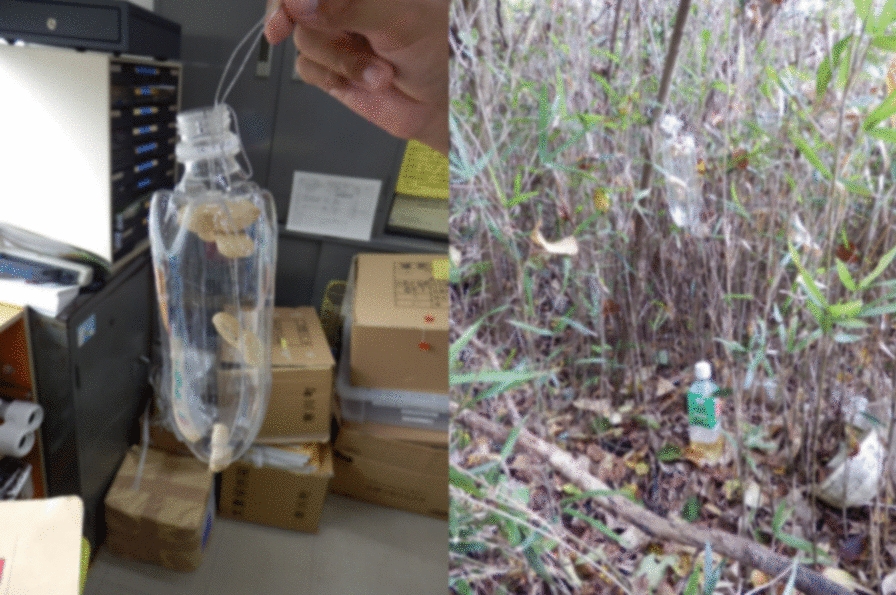
Photos of the bait trap. Close-up photo of the bait trap (left) and of the trap set in the field (right). If the top peanut has been taken, it is considered highly likely that a raccoon has visited the trap. Such traps are inexpensive, being made from plastic bottles, peanuts, and wires

The main implementation items in the raccoon management procedure in this department are shown in Table 3. Dissemination of information and awareness raising was implemented by providing information on a Web page, and via leaflets created by a municipal officer (Fig. 4). The bait trap involved a PET bottle trap that can be produced at low cost (Fig. 5). Traps were set by an officer and expert, and patrols were conducted by patrol teams. The monitoring and revision processes were conducted using camera traps, bait traps and trace surveys, and records of individual raccoons were collected (including sex, weight, any comments made by officers, etc.). This information was used as a reference for the next fiscal year, such as for establishing priority areas for capture. In addition, the raccoon distribution data was aggregated into a mesh at the prefectural level to grasp the distribution status. When necessary, an officer and expert exchanged information, including information about the situation in other areas. 20 traps were set over a period of about 7 months; from these traps, nine raccoons were captured in 2013 and 15 raccoons were captured in 2014.

The reference information available to officers is shown in Table 6.

**Table 6:** The reference information available to officers in each city

	Oita city	Asahikawa city	Hadano city
Referred sources	University	The Ministry of the Environment’s and Hokkaido regional manuals (Ministry of the Environment Hokkaido Regional Environment Office and EnVision Environmental Conservation Office [59, 39]	The Ministry of the Environment’s manual and Hokkaido’s technical guidelines (Hokkaido [53, 45]
Wanted to know	Indices to understand raccoon population and density; effective methods for raising awareness among residents	Raccoon ecology (e.g. longevity and regional characteristics); effective methods for capturing female	Raccoon ecology (e.g. regional characteristics); capture methods (bait, trap, and timing); trapping tools (effective, unbreakable, and cheap to buy)

### Challenges mentioned by the officers

Although local government in Oita city was able to manage raccoons through regional cooperation, together with the NPO and university, the difficulties gradually increased.

Officer: “The division does not possess a car for dealing with reports from residents, so it is impossible to go and check reports immediately, and more officers are needed to deal with increased reports and paperwork.”

Sighting reports increased about 1 year after management began, so that most of each day was spent on raccoon-related work, despite the fact that this was one of several tasks. Officers were not ready to deal with the large number of reports or implementing the trapping campaign.

Officer: “We are able to implement the trapping campaign in cooperation with residents in some places, but in other places this is difficult.”

Because of this difficulty, there are parts of the city in which the trapping campaign could not be conducted, and the same problem occurs at the prefectural level. Respondents mentioned the challenge that areas cannot be managed at a city-wide level, and instead are managed at the level of prefecture or country, which leads to the existence of unmanaged areas.

Respondents mentioned the challenge that, in some reports, residents make requests that are difficult to field. However, the environmental division is responsible for the management of non-native species, whereas the agricultural division is responsible for crop damage. This is why officers struggled to field requests such as:

Residents: “I want to manage animal damage by native species, why is it only raccoons that you are targeting?”

Budgets are divided according to the fiscal year. Therefore, it was difficult to respond flexibly to management requirements. For example, the unit prices of consumables and fixtures were specified in the budget. Cage traps are consumable items, and camera traps are fixtures. Because of budgetary restraints, unexpected requirements for consumables or fixtures were difficult to respond to.

There were three main challenges faced by the officers in Asahikawa city. Firstly, the management procedure could not be started during April and May, when racoon trapping is most cost effective due to their higher activity level during the breeding season. Secondly, it was difficult to publicize the results widely, and finally it was difficult to construct a management system after the completion of the funded project. Although the funded project was conducted over several years, it was necessary to establish a new control plan every year. It took some time for the new plans to be accepted, and trapping campaign was implemented from summer onwards. Moreover, reports were not published, and results were used only for presentations at workshops where the number of participants was limited. Regarding future issues:

Officer: “Although there are many tasks required to receive subsidies, once the subsidy program is finished, the burden of work may be reduced.”

Although a reduction of work burden may occur, it was mentioned that budget cuts may also cause reduced capture pressure.

Some other challenges were also raised. Regarding management procedures, although a meeting was held each year to make the next year plan, there were limited options that were reviewed. Finding signs of raccoon presence in forests and river environments that residents could not reach was mentioned as a challenge. A cooperative framework, requiring sharing of information among neighboring areas in different administrative districts, was mentioned as being necessary to understand the spatial distribution and damage caused by raccoons. It was pointed out that reports from residents that should be handled by other divisions were handled by trappers. This was because residents found it easy to report to trappers who were actively interviewed in the field. Moreover, other divisions that have no regularly employed staff recognized that crop damage countermeasures are part of their work but did not recognize the environmental aspects of the problem.

In Hadano city, one of the main challenges mentioned was that there were continuity problems when new officers were recruited.

Expert: “Successful racoon management is possible due to the excellent performance of the current officer, but the current system may not be able to continue if this officer changes”.

The current officer, having a hunting license and being accustomed to handling wildlife, conducted many of the required tasks, but was also responsible for work other than trap patrolling. In the next fiscal year, it was decided that fewer officers would be available for this project.

Officer: “Because staff numbers will decline, next year will be tough.”

The burden on officers was expected to increase, due to the increase in other responsibilities. It was mentioned that some work was requested at inexpensive rates due to low budget. Because reducing the cost of equipment is difficult, it was necessary to supplement the equipment budget with other parts of the budget, such as for labor. As a result, the patrols had almost no associated costs. When calculated in terms of salaries that would have been paid, the required budget was about five times the actual budget.

Officials mentioned that sharing of information with JAs was challenging. Agricultural cooperatives were responsible for crop damage countermeasures. Previously, damage reports did not mention whether raccoons were specifically responsible for damage, and reports were based only on the declaration of the farmer. Later, JAs telephoned the officer and confirmed the reports, although some reports were processed without being checked. Therefore, it was difficult to establish precise details about the number of trapping nights.

## Discussion

Although the management case study and experiences of managers are worth documenting [22, 41, 42], examples of management are difficult to publish [21]. By interviewing stakeholders, we successfully described the current management system and grasped the challenges faced by officers about raccoon management in Japan. We summarized the challenges faced by officers and the characteristics of management programs in Table 7. Our results indicate there is no simple solution that applies everywhere because the management systems, budgets, and challenges were complex and varied in each region. However, in raccoon management programs undertaken in many areas, information that helps to solve problems that occur in other areas is often available from other sources. For example, management in Asahikawa city became challenging as the budget was reduced, and this will continue to be a challenge in the future. The ideas developed in capture projects such as those carried out by Oita and Hadano cities can be used for this. Asahikawa city possesses the equipment necessary for management, such as the division of roles among stakeholders, on-site knowledge, and equipment. Furthermore, the municipality’s survey of the whole city reveals the approximate raccoon distribution and the location of high capture areas. Therefore, in this case, instead of distributing the effort evenly throughout the city, it is possible to adopt a strategy of focusing efforts in places where capturing and sightings are frequent, and by narrowing the range of the period, setting traps intensively, and lowering the density of raccoon.

**Table 7:** Summary of challenges faced by officers and characteristics of management programs

	Oita city	Asahikawa city	Hadano city
Main challenges faced by officers	Increased burden of tasks; cooperating with wide variety of residents (in neighboring areas); native mammal control; flexibility of budget	Subsidy system; decreased budget; small number of review options; cooperate with neighboring area; cooperating with agricultural division	Increased burden of tasks; Low budget; cooperating with Japan Agricultural Cooperatives
Characteristics of management	Positioning the raccoon problem as an environmental and biodiversity issue; cooperating with stakeholders; securing funding	Large scale and multiple year management with large budget; cooperating with diverse group of organizations; utilizing technical expertise	Small budget; management throughout the city, dividing it by area, and over different seasons; cooperating with experts

### Interpreting the three case studies

#### Common denominators of racoon management

Although the method of obtaining the budget was different for each municipality, there were two ways of increasing the budget. The first was to obtain funding within the organization, based on actual results. Steadily improving the control results, via collaboration between the government, private sector, and academics, depended on securing funding within the city’s budget. Administrative organizations in Japan can increase their budgets approved only if the results show improvements. The second was to obtain a subsidy from the national government.

Although we predicted that there might be a silver bullet that could progress management in many areas through the three case studies, we could not figure out. However, we offer three reasons to explain how efforts including monitoring were implemented, which can be used as a reference for solving the challenges. First is the common understanding that management should not start after damage (mostly to crops) has been reported. In many local government divisions, control is implemented only after damage to crops has occurred [30, 46]. There are two environmental divisions, which in our opinion are not mainly concerned with agricultural damage. The officers and experts have a cooperative relationship in which they are consider raccoon management as non-native species management. As a result of this awareness, it is thought that the department can gather information from the early stages of invasion, when little damage has occurred, and thereby assess the situation regardless of the magnitude of the damage.

The second reason we propose is that there is a desire for a collaborative approach. Although there were differences in the degree of sharing, all departments used a collaborative approach. In some interactions, the administration appealed to groups and organizations that were originally linked by other works, or were likely to have information about raccoon management; it also appealed to organizations involved in local environmental issues. Furthermore, because experts participated in these cooperative relationships, it is thought that (within departments) management and evaluation can occur before trapping activities are commenced.

The third reason we propose is that departments were able to select the methods and/or equipment appropriate for their budgets. For example, to assess the invasion status, a trade-off was required between the cost of traps and monitoring tools and their accuracy, while needing to understand the complexity of using a diverse array of monitoring and trapping methods throughout each city, including camera traps and trace surveys. Regarding our second and third proposals, cooperation with experts is essential; this will be discussed further.

We have obtained an understanding of the ideal raccoon management procedures. Although steps I, II, and III iv were implemented in several ways, steps III i–iii and v were, in practice, a single method. Step III i and v involved the planning and review of trapping sites and establishment of the timing; they were entirely focused on how to place cage traps. Step III ii mainly involved the provision of information and reporting by the administration, in the sense of fulfilling accountability requirements; there was no proactive process of establishing consensus between residents and officials. As for step III iii, although various people conducted the trapping, this was setting traps, patrolling, and collecting the raccoons that were caught. Practical techniques did not include kill traps or biological and chemical control methods. For example, kill traps and a sterilization vaccine have tried to apply during mongoose management in Japan [47, 48]. Because the acceptance among residents was high (Ikeda 2006), and there was no substantial opposition, step ii might be acceptable. However, steps III i and v require further consideration. It is clear that the planning and reviewing of trapping points and timing are important. It is known that raccoon foraging habits can vary from region to region [49], and that the waterfront is an important habitat element for racoons [24–26]. Therefore, it is necessary to select monitoring locations by taking known distributions into account. In general, it is recommended to trap immediately before breeding season [50, 51] so as to capture high-fertility individuals most effectively [50, 52]. In Hokkaido, capturing raccoons during spring is most efficient because the raccoon birthing-to-weaning period is roughly from March to July; young juveniles cannot survive when the mother is captured before they are weaned (Hokkaido [38, 53]. However, there are still some factors to be reviewed. For example, it is not well known how native species are affected by raccoons. It is also possible to examine management procedures by considering native species that may be affected by raccoons, and their habitats; these include arthropods, amphibians, reptiles, birds, and mammals. In addition, it is known that raccoon populations in North America selectively eliminate female adults with high fertility, to reduce population density [54]. In Japan, the development of technology to selectively capture female raccoons is under way [47], and it is considered to be an option for practical use.

### Support from experts

In conservation, it is widely accepted that there is a large gap between researcher priorities and the demands of practitioners [17–20]. To fill this gap, it is said that researchers and practitioners need to cooperate closely, from basic research through to implementation [17, 19, 55]. In the case of our study, the officers ranged from people closely associated with wildlife, such as hunters, to people not at all familiar with wildlife, who cooperate with researchers or experts. People with expert knowledge contributed to the revision of management plans, survey methods, and interpretation of results, and essentially cooperated beyond the fiscal year. They cooperated not only during a one-shot workshop but also in the first stages of management such as the planning stage. The roles of the experts required varied according to region and situation. Depending on how experts are involved, they can contribute to the analysis of captured individuals to figure out effective season for trapping, as in Takatsuki et al. [49]. For the selection of methods such as a trace surveys which need accuracy, expert assistance is considered important, particularly when budgets are low.

The interpretation of management outcomes and review of methods are important aspects of scientific management; they are necessary for administrations to fulfill accountability requirements. Local government departments struggle to analyze data and apply it in future; however, the administration can record the capture status, identity of individual raccoons, and regional data. Currently, the indicators used to measure the effectiveness of raccoon management are mainly the extent of crop damage, the capture effort, the number of captures, and capture per unit effort (CPUE), which is calculated from the two latter indicators. However, these indicators are difficult to apply in regions where it is impossible to record capture effort. This challenge exists in regions that have just started managing racoons, where it is necessary to achieve trap nights in excess of 1000 before indicators can be calculated. Regarding crop damage, there are also problems with the accuracy of reports, in terms of the judgment of and declarations made by agricultural workers. In addition to the problems associated with such indices, even if the estimated density is obtained, there are few options for reviewing such data, and the best option may be to continue capture. Researchers and experts need to cooperate in reviewing appropriate methods for each area, and in developing indices that can be evaluated by the available staff.

### Information required by officers

The officers who were actively managing raccoons used information from experts and management manuals (guidelines) from both the Ministry of the Environment and each local government. The manuals were mostly from the Ministry of the Environment [45], the local office of the Ministry of the Environment (e.g. Ministry of the Environment, Kinki local office [40], prefectures (e.g. Oita [56]. They used data that were compiled from trapping campaigns and/or from projects that studied invasion status. The ecology of raccoons was generally presented consistently in all the manuals. The manuals differed in terms of the other information presented, such as the amount of explanation provided. The Ministry of the Environment’s manual provides substantial information on the conceptual aspects of control, and on procedures and methods, but each prefectural manual tends to contain information on the current invasion status of the area. From reading these, an officer can understand the fundamental ecology and approach required. Reading one manual alone is considered insufficient to give officers and residents an understanding of the management concepts and practices in the region. Indeed, as pointed out by one officer, “I had to fumble my way at first”; this is from an officer who had read two kinds of manuals. Knowledge about management requires practice, because on-site knowledge and case studies are not described in detail in the manuals. One manual (Ministry of the Environment, Kinki local office 2008) mentions a case study. However, it is limited to the introduction of the management system. Even during the interaction with the officer in our interview surveys, there was a case in which an officer inquired about basic raccoon ecology, which is mentioned briefly in the manuals. This is consistent with the findings of Matzek et al. [19]. In addition, in terms of information that the officers wanted to know, they mentioned information that could be utilized on site, such as effective methods to motivate residents, as well as information about ecology and capture methods. These officers wanted to know about the ecology in other areas from people involved in management, and about their impressions during field work. Based on our survey of officers trying to implement management practices, it is necessary to consider how to provide the information that officers require, and that is not provided in the manuals.

## Conclusions

Ikeda [41] and the Ministry of the Environment [42] have pointed out the necessity of sharing information for successful racoon management. Accumulating cases of management failures, in terms of invasive nonnative species management, and analyzing such problems, is indispensable for implementing future management programs more effectively [57, 58]. It is important to share lessons about failed programs and efforts. Regardless of the area in which management is being promoted (prefecture or municipality), or whether the invasion is severe, the problem remains that officers find it “difficult to understand the present status of the raccoon in the area (such as number of individuals, density, and distribution) [11] ”. Officers used integrating reports and camera trap records, and conducted intensive trapping and control programs. By placing camera traps and traps throughout the city, officers were able to assess the approximate distribution of raccoons, within a funded project. Officers focused on trace surveys and bait traps, while also using camera traps, and they were able to assess the distribution and implement trapping and control. While these methods and the flow of efforts have advantages and disadvantages—for example, bait traps are cheap but less effective in places with abundant bait resources [45]—methods are available that are relatively inexpensive, and that do not require expert skills. This information can be referred to in areas where budgets are low, and where programs are starting with small budgets and without staff who have specialized knowledge. Challenges may change according to the progress of each region and management. We will be able to provide useful information to inform management by understanding the type of information that stakeholders require, and by collecting and organizing case studies about the challenges that they experience.

## Data Availability

The datasets used and/or analyzed during the current study are available from the corresponding author on reasonable request.
